# Prediction of oxygen uptake at peak exercise in patients with inflammatory bowel disease using submaximal cardiopulmonary exercise testing and the steep ramp test as a practical field test

**DOI:** 10.1371/journal.pone.0354707

**Published:** 2026-10-08

**Authors:** Karlijn Demers, Sander M. J. van Kuijk, Marieke J. Pierik, Daisy M. A. E. Jonkers, Laurents P. S. Stassen, Bart C. Bongers

**Affiliations:** 1 . Department of Surgery, Maastricht University Medical Center+, Maastricht, the Netherlands; 2 . Department of Gastroenterology-Hepatology, Maastricht University Medical Center+, Maastricht, the Netherlands; 3 . Department of Surgery, Institute of Nutrition and Translational Research in Metabolism (NUTRIM), Maastricht University, Maastricht, the Netherlands; 4 . Department of Gastroenterology-Hepatology, Institute of Nutrition and Translational Research in Metabolism (NUTRIM), Maastricht University, Maastricht, the Netherlands; 5 . Department of Clinical Epidemiology and Medical Technology Assessment (KEMTA), Maastricht University Medical Center+, Maastricht, the Netherlands; 6 . Department of Nutrition and Movement Sciences, Institute of Nutrition and Translational Research in Metabolism (NUTRIM), Maastricht University, Maastricht, the Netherlands; University of Hertfordshire, UNITED KINGDOM OF GREAT BRITAIN AND NORTHERN IRELAND

## Abstract

**Purpose:**

To develop predictive equations for oxygen uptake at peak exercise (VO_2peak_) measured during a cardiopulmonary exercise test (CPET) for patients with inflammatory bowel disease (IBD) using submaximal CPET-derived parameters and the steep ramp test, along with demographic and clinical characteristics.

**Methods:**

This study included patients with IBD in remission or with mild-to-moderate disease activity who performed a CPET and a steep ramp test within 14 days. Submaximal CPET-derived parameters included oxygen uptake (VO_2_) at the ventilatory anaerobic threshold (VAT) and oxygen uptake efficiency slope (OUES), while the outcome of the steep ramp test was the work rate achieved at peak exercise (WR_peak_).

**Results:**

Predictive regression equations with submaximal VO_2_ at the VAT and with OUES, when combined with age and body fat percentage, explained >89% of the variance in VO_2peak_ (optimism-adjusted R^2^ = 0.895 and 0.938). Predictive capacity increased when both VO_2_ at the VAT and OUES were combined in a predictive regression equation, together with age, body fat percentage, and clinically active disease (optimism-adjusted R^2^ = 0.951). Steep ramp test WR_peak_ explained 89% of the variance in VO_2peak_ (optimism-adjusted R^2^ = 0.892).

**Conclusion:**

The highly predictive equations developed in this study support the use of the submaximal CPET and the steep ramp test as alternatives for assessing VO_2peak_ in patients with IBD in remission or with mild-to-moderate disease activity in clinical and research settings.

## Introduction

Cardiorespiratory fitness, representing the body’s capacity to deliver and utilize oxygen during large muscle physical activity, is a well-established indicator of overall health and is strongly associated with morbidity and mortality in the general population [1]. In individuals with inflammatory bowel disease (IBD), improved cardiorespiratory fitness may positively influence subjective well-being, mitigate mucosal inflammation, or reduce the risk of adverse postoperative outcomes, but evidence remains limited [2].

Routine assessment of cardiorespiratory fitness is crucial to understand its clinical implications in IBD and identifying potential therapeutic avenues that could enhance patient outcomes through targeted physical exercise interventions. The gold standard test for assessing cardiorespiratory fitness involves quantifying oxygen uptake at peak exercise (VO_2peak_) during a cardiopulmonary exercise test (CPET), requiring maximal exertion. However, in certain patients with IBD, maximal exertion may be constrained by disease-specific factors, such as disease activity, abdominal pain, fatigue, arthralgia, or psychological barriers such as lack of motivation or the fear of exacerbating symptoms [3,4]. In such cases, submaximal CPET-derived metrics, including the oxygen uptake (VO_2_) at the ventilatory anaerobic threshold (VAT) and the oxygen uptake efficiency slope (OUES), may provide valid alternatives for evaluating cardiorespiratory fitness [5].

Despite CPET being the most accurate method for evaluating cardiorespiratory fitness, its use in clinical settings is often constrained by relatively high costs, time requirements, and the need for specialized personnel and equipment [1]. The steep ramp test, a practical, short maximal exercise test performed on a cycle ergometer that does not require respiratory gas analysis, could serve as a less demanding alternative [6]. In a previous study, a very strong correlation was found between the outcome of the steep ramp test, *i.e.,* the work rate achieved at peak exercise (WR_peak_), and VO_2peak_ attained during CPET [7].

To evaluate the possibility to use the submaximal CPET and the steep ramp test in clinical and research settings for patients with IBD in remission or with mild-to-moderate disease activity, and to enable comparisons with population-based normative data, the objective of this study was to develop predictive equations for VO_2peak_ measured with CPET, using (1) submaximal CPET-derived parameters (*i.e.,* VO_2_ at the VAT, OUES) and (2) steep ramp test performance (WR_peak_), both combined with demographic and clinical characteristics.

## Methods

### Study design and participants

Adult patients (≥18 years) with Crohn’s disease (CD) or ulcerative colitis (UC) were recruited for this cross-sectional study from the gastroenterology outpatient clinic between August 17, 2022, and November 11, 2023. Detailed in- and exclusion criteria are provided elsewhere. In brief, eligible patients were required to be in remission or have mild-to-moderate clinical disease activity (Harvey Bradshaw index ≤16 or simple clinical colitis activity index ≤11) [8,9]. Patients were excluded if they had injuries or comorbidities potentially affecting study evaluations, or if they were pregnant, lactating, or competitive/elite athletes. This study was conducted in compliance with the Declaration of Helsinki and was approved by the Medical Ethical Committee. All patients provided written informed consent.

### Data collection

Demographic and clinical data from all participants was collected during the study visit and from electronic patient records, including age, sex, Charlson comorbidity index, smoking status, disease duration, medication use, and prior intestinal resections. Active clinical disease at time of inclusion was defined as a Harvey Bradshaw ≥5 for patients with CD or a simple clinical colitis activity index of ≥3 for patients with UC. Faecal calprotectin values, if available within 8 weeks of inclusion, were collected, with levels below 250 µg/g indicating biochemical remission.

### Anthropometry and exercise testing

Body weight and height were measured barefoot and wearing light clothing to calculate body mass index (BMI). Body fat percentage was estimated by calculating the cumulative sum of four skinfold thicknesses (*i.e.,* biceps, triceps, subscapular, and suprailiac) and applying it to the age- and sex-specific prediction equation by Durnin and Womersley [10].

The CPET and steep ramp test were conducted on two separate days, with a maximum interval of 14 days between tests. Detailed testing protocols are described elsewhere [7]. Resting baseline values were recorded before each test, and rate of perceived exertion was evaluated pre- and post-test using the 6–20 Borg scale [11]. The CPET on a cycle ergometer (Lode Corival CPET, Lode BV, Groningen, the Netherlands) started with a 3-minute warm-up of unloaded cycling, followed by an incremental increase in work rate *(i.e.,* resistance or cycling load, expressed in Watts*)* tailored to the participant's estimated fitness level, aiming for maximal effort within 8–12 minutes. Participants were instructed to maintain a pedaling frequency of 70–80 rpm, with the test terminating when frequency fell below 60 rpm, indicating the point of maximal exertion. Peak exercise parameters were averaged over the final 30 seconds before this point. During the test, participants wore an oro-nasal facemask connected to a calibrated ergospirometry system for respiratory gas analysis (Vyntus CPX four, Vyaire Medical, Mettawa, IL, USA). Heart rate and rhythm were monitored using a 12-lead electrocardiogram (Vyntus ECG 12-lead PC-ECG, Vyaire Medical, Mettawa, IL, USA). SpO_2_ was continuously measured, and blood pressure (Tango M2 Stress Test Monitor, SunTech Medical Inc., Morrisville, NC, USA) was measured every 2 minutes. VO_2peak_ was considered (near-)maximal when the respiratory exchange ratio (RER_peak_) exceeded 1.10 and/or when heart rate at peak exercise (HR_peak_) surpassed 85% of the predicted maximum (*i.e., 208 – 0.7 × participant’s age*). Additionally, participants had to show subjective signs of maximal effort. The VAT was defined as the point of an increase in carbon dioxide production relative to the oxygen uptake (VO_2_) using the V-slope method. This point was confirmed by marking where the ventilatory equivalent for oxygen and partial end-tidal oxygen tension began to consistently increase after reaching a minimum, while the ventilatory equivalent for carbon dioxide and partial end-tidal carbon dioxide tension remained constant. The OUES was calculated based on the linear relationship between VO_2_ and the logarithm of minute ventilation throughout exercise, using all available test data from the beginning of the work rate increments to the point of test termination or up to a clear VO_2_ plateau, when applicable. CPET interpretation was conducted by a trained and experienced clinical exercise physiologist (BB) and subsequently verified by a medical doctor trained in clinical exercise physiology (KD) using a systematic, guideline-based approach [12]. Any discrepancies were reviewed and discussed until a consensus was reached.

The steep ramp test, performed on a cycle ergometer started with a 3-minute warm-up of unloaded cycling, followed by an incremental work rate increase of 25 W every 10 seconds (2.5 W/s). Participants cycled until exhaustion at a pedaling frequency of 70–80 rpm. WR_peak_, normalized to body mass, was defined as the work rate at the point at which the pedaling frequency dropped below 60 rpm despite strong verbal encouragement. Heart rate and peripheral oxygen saturation were continuously recorded.

### Statistical analyses

Categorical variables were presented as absolute number and percentage, while continuous variables were described as median with the first and third quartile (Q1, Q3). Differences between the CPET and steep ramp test values were assessed using the paired-samples t-test for normally distributed data and Wilcoxon signed-rank test when normality assumptions were violated, or outliers were present. To develop predictive equations for VO_2peak_, univariable and multivariable linear regression analyses were used. Initially, predictive regression equations were developed including test outcomes only. Subsequently, demographic and clinical factors were added to the model to assess if predictive accuracy improved. Univariable linear regression analyses were used to assess the association between VO_2peak_ and individual demographic and clinical factors. Variables with a *p* < 0.10 in the univariable analyses were included in the multivariable models, along with submaximal CPET-derived parameters (*i.e.,* VO_2_ at the VAT and OUES) and steep ramp test WR_peak_. Backward elimination on the Wald test was used for the multivariable models was used. The predictive equations were derived from the final models, and the apparent-performance was quantified as R^2^ to assess the models’ explanatory power. To estimate performance in future patients (*i.e.,* patients who were not used to derive the formulae), the predictive models were internally validated through bootstrapping with 1000 bootstrap samples to calculate the optimism-adjusted performance. This optimism-adjusted R^2^ provides a more realistic estimation of the models’ performance when applied to new patients from the same underlying population. All statistical analyses were performed using IBM SPSS Statistics and R statistical software. Statistical significance was defined as *p* < 0.05.

## Results

A total of 53 patients (*n* = 29 CD, *n* = 24 UC) were included in this study, with their demographic and clinical characteristics summarized in Table 1. The median age of the study population was 42.7 years, with 45.3% being female. Exercise test outcomes for the CPET and the steep ramp test are presented in Table 2. One participant was unable to deliver a maximal effort during the CPET and was therefore excluded from VO_2peak_ analyses. The steep ramp test had a significantly shorter test duration compared to the CPET (*p* < 0.001), while WR_peak_ was significantly higher during the steep ramp test (*p* < 0.001). Additionally, patients achieved a higher HR_peak_ during the CPET (*p* < 0.001), and the change in rate of perceived exertion before and after the test was significantly greater for the CPET compared to the steep ramp test (*p* = 0.038).

**Table 1:** Demographic and clinical characteristics of included patients.

Demographic and clinical characteristics	Total (*n* = 53)
Age at inclusion (years), *median (Q1, Q3)*	42.7 (32.2, 54.3)
Sex, female, *n (%)*	24 (45.3)
BMI (kg/m^2^), *median (Q1, Q3)*	25.2 (23.2, 28,1)
CCI, *n (%)*	
0	31 (58.5)
1–2	19 (35.8)
>2	3 (5.7)
Disease phenotype, *n (%)*	
CD	29 (54.7%)
UC	24 (45.3%)
Disease duration (years), *median (Q1, Q3)*	11.3 (7.0, 22.7)
Montreal age at diagnosis, *n (%)*	
A1: ≤16 years	7 (13.2)
A2: 17–40 years	36 (67.9)
A3: >40 years	10 (18.9)
Montreal disease location [CD], *n (%)*	
L1: ileal	10 (34.5)
L2: colonic	4 (13.8)
L3: ileocolonic	15 (51.7)
+ Perianal disease	7 (13.2)
+ Upper gastrointestinal disease	3 (10.3)
Montreal disease behavior [CD], *n (%)*	
B1: non-stricturing, non-penetrating	14 (48.3)
B2: stricturing	6 (20.7)
B3: penetrating	9 (31.0)
Montreal disease extension [UC], *n (%)*	
E1: proctitis	1 (4.2)
E2: left-sided colitis	9 (37.5)
E3: pancolitis	14 (58.3)
Clinical disease activity ^a^, *n (%)*	
Remission	40 (75.5)
Mild disease activity	12 (22.6)
Moderate disease activity	1 (1.9)
Fecal calprotectin^b^ (μg/g), *median (Q1, Q3)*	38 (16, 158)
Biochemical disease activity^b^, *n (%)*	
Remission	41 (85.4)
Active disease	7 (14.6)
Current smoking status, *n (%)*	
Smokes daily	4 (7.5)
Smokes occasionally	3 (5.7)
Ex-smoker	21 (39.6)
Never smoked	25 (47.2)
Current IBD medication*, n (%)*	
None	10 (18.9)
Mesalazine only	5 (9.4)
(Topical) corticosteroids	2 (3.8)
Immunomodulators	5 (9.4)
Biologic agents	31 (58.5)
Prior intestinal resection, *n (%)*	12 (22.6)

Abbreviations: CCI = Charlson comorbidity index, CD = Crohn’s disease, HBI = Harvey Bradshaw index, IBD = inflammatory bowel disease, Q1 = 1^st^ quartile, Q3 = 3^rd^ quartile, SCCAI = simple clinical colitis activity index, UC = ulcerative colitis.

^a^Clinical disease activity according to the HBI for CD and the SCCAI for UC: remission was defined as HBI <5 or SCCAI <3, mild disease activity as HBI 5–7 or SCCAI 3–5, and moderate disease activity as HBI 8–16 or SCCAI 6–11;

^b^Available in *n* = 48; remission was defined as faecal calprotectin <250 ug/g;

**Table 2:** Exercise test outcomes for CPET and the steep ramp test.

Exercise test outcomes	CPET	Steep ramp test	*p*
Test duration^b^ (s)	597.0 (539.0, 627.5)	126.0 (112.0, 152.4)	***<*0.001** ^**a**^
WR_peak_ (W)	199 (168, 250)^c^	315 (280, 381)	***<*0.001** ^**a**^
WR_peak_ (W/kg)	2.8 (2.1, 3.5)^c^	4.1 (3.5, 5.0)	***<*0.001** ^**a**^
HR_peak_ (bpm)	177.5 (163.5, 190.8)	167.0 (157.5, 178.5)	***<*0.001** ^**a**^
Δ 6-20 Borg RPE	9.0 (7.0, 11.0)	8.0 (6.0, 9.5)	**0.038** ^**a**^
VO_2peak_ (mL/kg/min)	32.0 (25.2, 38.5)^c^	N/A	N/A
VO_2_ at the VAT (mL/kg/min)	16.4 (14.1, 20.0)	N/A	N/A
OUES/kg	31.8 (26.2, 38.9)	N/A	N/A
RER_peak_	1.2 (1.2, 1.3)^c^	N/A	N/A
VE_peak_ (L/kg)	1.3 (1.0, 1.5)^c^	N/A	N/A
O_2_-pulse (mL/beat)	13.9 (11.0, 16.6)^c^	N/A	N/A
BF_peak_ (breaths/min)	39.1 (35.0, 43.6)^c^	N/A	N/A
VE/VCO_2_ ratio at the VAT	26.3 (24.5, 28.3)	N/A	N/A
VE/VCO_2_-slope	27.2 (25.4, 29.7)	N/A	N/A

Abbreviations: BF_peak_ = breathing frequency at peak exercise, HR_peak_ = heart rate at peak exercise, N/A = not applicable, O_2_-pulse = oxygen pulse, OUES = oxygen uptake efficiency slope, RER_peak_ = respiratory exchange ratio at peak exercise, RPE = rate of perceived exertion, VAT = ventilatory anaerobic threshold, VE_peak_ = minute ventilation at peak exercise, VCO_2_ = carbon dioxide production, VO_2_ = oxygen uptake, VO_2peak_ = oxygen uptake at peak exercise, WR_peak_ = work rate at peak exercise.

^a^statistically significant *p*  <  0.05

^b^duration of the ramp phase, excluding warming-up and cooling-down.

^c^available in *n* = 52, as one participant was unable to reach maximal effort during CPET

The linear associations of VO_2_ at the VAT, OUES, and steep ramp test WR_peak_, with VO_2peak_ are shown in Fig 1 (A-C). Also, an acceptable construct validity was found for the steep ramp test in predicting submaximal CPET VO_2_ at the VAT and OUES (R^2^ = 0.68 and R^2^ = 0.74, respectively; S1 Fig in S1 File). Linear regression analyses produced the following predictive equations to predict VO_2peak_ based on test outcomes only, including (1) VO_2_ at the VAT, (2) OUES, (3) VO_2_ at the VAT and OUES combined, and (4) steep ramp test WR_peak_.

**Fig 1:**
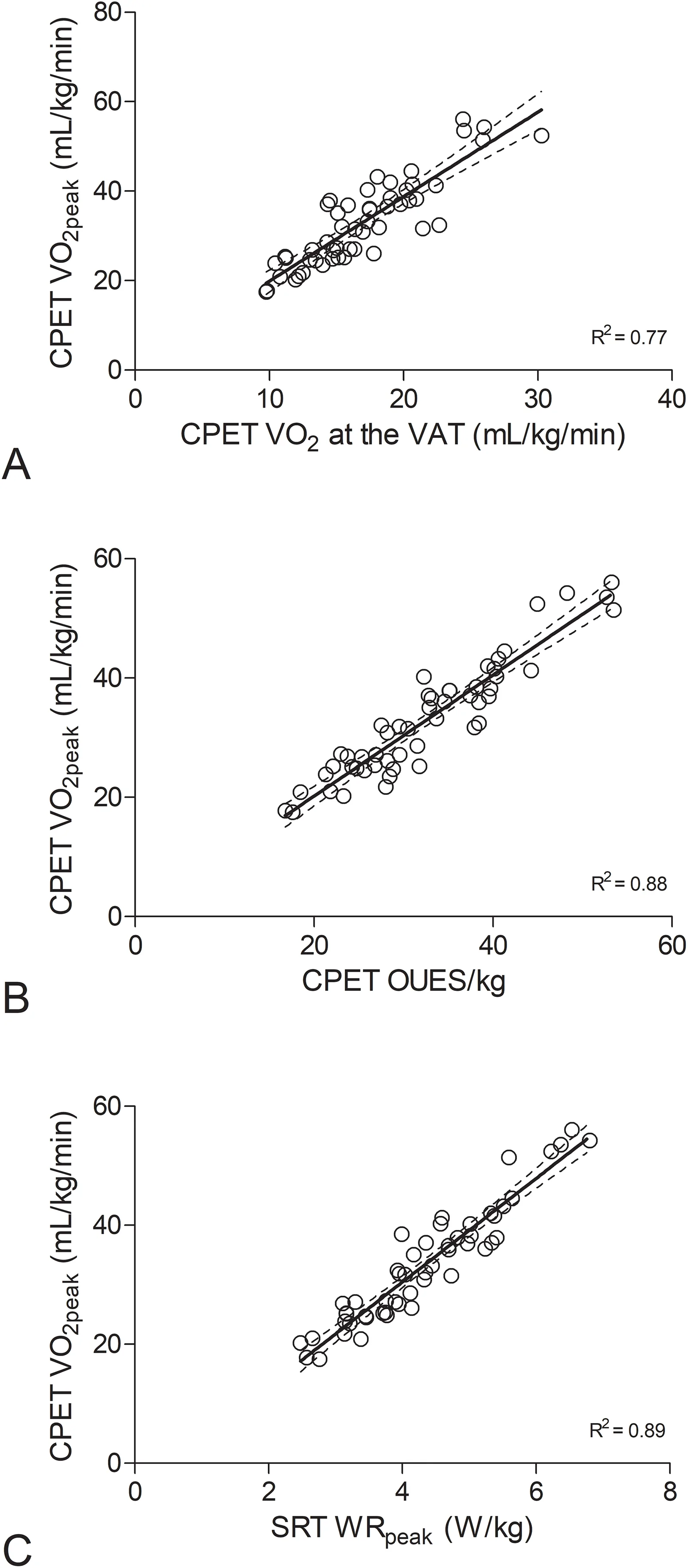
Linear relationships of CPET VO_2peak_ with submaximal CPET-derived parameters and the steep ramp test. Plots showing the best fit-line with 95% confidence intervals between CPET VO_2peak_ and (A) VO_2_ at the VAT, (B) OUES, and (C) steep ramp test WR_peak_. Abbreviations: OUES = oxygen uptake efficiency slope, SRT = steep ramp test, VAT = ventilatory anaerobic threshold, VO_2peak_ = oxygen uptake at peak exercise, WR_peak_ = work rate at peak exercise.

(1) Predicted VO_2peak_ [mL/kg/min] = 1.033 + (1.888 × VO_2_ at the VAT [mL/kg/min]) *(optimism-adjusted R*^*2*^ *= 0.761)*
(2) Predicted VO_2peak_ [mL/kg/min] = −0.092 + (1.016 × OUES/kg) *(optimism-adjusted R*^*2*^ *= 0.876)*
(3) Predicted VO_2peak_ [mL/kg/min] = −1.480 + (0.529 × VO_2_ at the VAT [mL/kg/min]) + (0.783 × OUES/kg) *(optimism-adjusted R*^*2*^ *= 0.887)*
(4) Predicted VO_2peak_ [mL/kg/min] = −4.421 + (8.718 × steep ramp test WR_peak_ [W/kg]) *(optimism-adjusted R*^*2*^ *= 0.892)*

Associations between demographic and clinical factors with VO_2peak_ are presented in S1 Table in S1 File. Multivariable linear regression analyses incorporating demographic and clinical factors alongside test outcomes revealed prediction equations for VO_2peak_ based on (1) VO_2_ at the VAT, (2) OUES, and (3) their combination. Adding demographic and clinical factors made no statistically significant contribution to the steep ramp test model. The apparent-performance and optimism-adjusted R^2^ values for each predictive equation are available in S2 Table in S1 File.

(1) Predicted VO_2peak_ [mL/kg/min] = 29.660 + (−0.092 × age [years]) + (−0.447 × body fat [%]) + (1.227 × VO_2_ at the VAT [mL/kg/min]) *(optimism-adjusted R*^*2*^ *= 0.895)*
(2) Predicted VO_2peak_ [mL/kg/min] = 20.931 + (−0.098 × age [years]) + (−0.281 × body fat [%]) + (0.761 × OUES/kg) *(optimism-adjusted R*^*2*^ *= 0.938)*
(3) Predicted VO_2peak_ [mL/kg/min] = 18.967 + (−0.098 × age [years]) + (−0.254 × body fat [%]) +(−1.455 × active clinical disease) + (0.509 × VO_2_ at the VAT [mL/kg/min]) + (0.542 × OUES/kg) *(optimism-adjusted R*^*2*^ *= 0.951)*

## Discussion

Since the assessment of VO_2peak_ during CPET can be unfeasible or impractical for patients with IBD in clinical and research settings, this study developed predictive equations for VO_2peak_ in patients with IBD in remission or with mild-to-moderate disease activity, based on submaximal CPET-derived parameters (*i.e.,* VO_2_ at the VAT and OUES) and the steep ramp test WR_peak_. The current findings showed that both submaximal CPET-derived parameters, when combined with age, body fat percentage, and the presence of clinically active disease, and the steep ramp test, showed strong predictive accuracy for estimating VO_2peak_, explaining over 89% of the variance.

Submaximal CPET parameters, such as VO_2_ at the VAT and OUES, are well-established indicators of cardiorespiratory fitness and have demonstrated strong correlations with VO_2peak_, especially in populations where achieving maximal effort may be constrained [5,13–16]. VO_2_ at the VAT reflects the transition from mainly aerobic metabolism to a significant contribution of anaerobic metabolism as an additional energy source, whose relative contribution to total energy production increases with further increasing exercise intensity [5,17]. Hence, the VO_2_ at the VAT provides a physiologically meaningful threshold related to cardiorespiratory fitness. The OUES represents the ventilatory efficiency with respect to VO_2_ during progressive exercise [18]. In this study, the combination of VO_2_ at the VAT and OUES, when combined with age, body fat percentage, and the presence of clinically active disease, accounted for 95% of the variance in VO_2peak._ This high predictive accuracy underscores the suitability of these measures for assessing cardiorespiratory fitness, particularly in patients whose performance may be limited by factors other than cardiorespiratory deficiency. Potential arguments for avoiding maximal exertion in patients with IBD may include fatigue, joint pain, severe disease activity, prior to a surgical intervention, or psychological concerns, such as fear of symptom exacerbation during maximal effort. While one patient was unable to achieve maximal VO_2peak_ in the current study, all participants successfully reached the VAT. The high success rate of achieving VO_2peak_ in the current population could be explained by the fact that the cohort consisted of patients in remission or with only mild-to-moderate disease activity, excluding those with severe disease activity. Furthermore, patients with significant joint complaints, such as active arthritis, were excluded.

In outpatient or resource-constrained settings, the steep ramp test could serve as a practical alternative to the CPET in patients with IBD, in which the achieved WR_peak_ explained 89% of the variance in VO_2peak._ High reliability of the steep ramp test was also found in other populations [6]. A key advantage of the steep ramp test is that it does not require expensive respiratory gas analysis and specialized personnel, making it more accessible for routine clinical practice or research settings. Furthermore, the current findings highlight that the steep ramp test imposes a significantly lower burden on patients compared to CPET, with shorter test durations, lower heart rates at peak effort, and a smaller change in rate of perceived exertion after the test. These characteristics may make the steep ramp test also appropriate for use in patients with more advanced disease activity of severe fatigue. However, its applicability in patients with severe disease activity requires further investigation. Further, the addition of demographic and clinical variables to the regression model did not significantly enhance its predictive accuracy, indicating that the steep ramp test alone provides sufficient accuracy for estimating VO_2peak_.

Although a large body of evidence highlights the importance of cardiorespiratory fitness as a critical health indicator, its potential role as a modulator of disease and postoperative outcomes in patients with IBD remains poorly evaluated. This study addresses an important gap by contributing to the integration of less-invasive routine assessments of cardiorespiratory fitness into clinical practice and research initiatives for patients with IBD. The developed regression equations for estimating VO_2peak_ using CPET-derived parameters and the steep ramp test facilitate comparisons with normative values from healthy populations, offering a method to identify cardiorespiratory deficiencies in patients with IBD. Future research should focus on identifying effective therapeutic strategies to improve cardiorespiratory fitness in patients with IBD and investigate whether these improvements contribute to better disease control, including reduced inflammation and improved patient-reported outcomes, as well as improved postoperative outcomes.

A limitation of the study is the relatively small sample size of 53 patients, which may limit the precision of its regression coefficient estimates. Although the regression models demonstrated strong predictive accuracy after internal validation, larger, multicenter studies are needed to externally validate these equations in more extensive and diverse IBD populations. Moreover, the exclusion of patients with severe disease activity may lead to differences in the predictive accuracy of the models when applied to patients with severely active disease. Another limitation is the potential variability in the determination of the VAT. While previous research indicates that the inter-observer variability of VAT measurement is acceptable for experienced clinicians, it is important to note that the VAT can sometimes be challenging to identify [19–21].

## Conclusion

In this study, we developed highly predictive equations for estimating VO_2peak_ in patients with IBD in remission or with mild-to-moderate disease activity using submaximal CPET-derived parameters and the steep ramp test, combined with demographic and clinical characteristics. These methods offer more feasible and practical alternatives to the traditional maximal CPET, making the assessment of cardiorespiratory fitness more accessible in clinical and research settings for patients with IBD. This could improve the identification of patients with low cardiorespiratory fitness levels and aid in the development of personalized physical exercise training interventions, potentially contributing to better disease management and enhanced quality of life for individuals with IBD.

## Supporting information

S1 File Supplementary figure and tables. This file contains S1 Fig, showing the construct validity of the steep ramp test in comparison with submaximal CPET-derived parameters; S1 Table, presenting the outcomes of the univariate linear regression analyses; and S2 Table, presenting regression equations to predict CPET VO_2peak_ based on submaximal CPET parameters and steep ramp test performance in patients with inflammatory bowel disease.
(DOCX)
