# Supplementary material for: Prediction of oxygen uptake at peak exercise in patients with inflammatory bowel disease using submaximal cardiopulmonary exercise testing and the steep ramp test as a practical field test
Source: PLoS One. 2026 Oct 8;21(10):e0354707. doi: 10.1371/journal.pone.0354707 (PMC13649012; doi:10.1371/journal.pone.0354707)
Supplement: Supplementary file 1 [file pone.0354707.s001.docx]

**Supplementary materials (S1 File)**

**S1 Fig. Construct validity of the steep ramp test in comparison to submaximal CPET-derived parameters.**

Plots showing the best fit-line with 95% confidence intervals between steep ramp test WR_peak_ and (A) VO_2_ at the VAT, and (B) the OUES. Abbreviations: OUES = oxygen uptake efficiency slope, VAT = ventilatory anaerobic threshold, VO_2_ = oxygen uptake, WR_peak_ = work rate at peak exercise.

**S1 Table. Outcomes of univariate linear regression**

| Variables | CPET VO_2peak_ (mL/kg/min) | | |
| --- | --- | --- | --- |
|  | **B** | **95% CI** | ***p*** |
| Age at inclusion (years) | -0.309 | -0.470, -0.147 | **<0.001^a^** |
| Male sex | 8.749 | 3.865, 13.633 | **<0.001 ^a^** |
| BMI (kg/m^2^) | -1.198 | -1.719, -0.676 | **<0.001 ^a^** |
| Body fat (%) | -0.932 | -1.102, -0.761 | **<0.001 ^a^** |
| CCI score | -4.263 | -6.911, -1.615 | **0.002 ^a^** |
| UC disease phenotype | 4.809 | -0.519, 10.137 | 0.076 |
| Disease duration (years) | -0.298 | -0.566, -0.030 | **0.030 ^a^** |
| Clinical disease activity^b^ | -6.493 | -12.708, -0.277 | **0.041 ^a^** |
| Biochemical disease activity | 4.974 | -2.828, 12.775 | 0.206 |
| Calprotectin value (ug/g) | 0.003 | -0.006, 0.011 | 0.544 |
| Active smoking | -3.369 | -11.316, 4.577 | 0.398 |
| Current corticosteroid medication | -0.874 | -9.127, 7.379 | 0.832 |
| Current biologic medication | -2.177 | -7.671, 3.318 | 0.430 |
| Prior intestinal resection | -6.579 | -12.788, -0.371 | **0.038 ^a^** |

Abbreviations: BMI = body mass index, CCI = Charlson comorbidity index, CI = confidence interval, CPET = cardiopulmonary exercise test, UC = ulcerative colitis.

^a^ significant *p* < 0.05

^b^ clinical disease activity according to the Harvey Bradshaw index for CD (score 5 or higher) and the simple clinical colitis activity index for UC (score 3 or higher)

**S2 Table. Regression equations to predict CPET VO_2peak_ based on submaximal CPET parameters (with and without additional demographic and clinical characteristics) and steep ramp test performance in patients with IBD**

| Regression equations | Apparent-performance  R^2^ | Optimism-adjusted R^2^ |
| --- | --- | --- |
| *Submaximal CPET parameters* |  |  |
| (1) VO_2peak_ [mL/kg/min] = 1.033 + (1.888 × VO_2_ at the VAT [mL/kg/min]) | 0.769 | 0.761 |
| (2) VO_2peak_ [mL/kg/min] = -0.092 + (1.016 × OUES/kg) | 0.879 | 0.876 |
| (3) VO_2peak_ [mL/kg/min] = -1.480 + (0.529 × VO_2_ at the VAT [mL/kg/min]) + (0.783 × OUES/kg) | 0.893 | 0.887 |
| (4) VO_2peak_ [mL/kg/min] = 29.660 + (-0.092 × age [years]) + (-0.447 × body fat [%]) + (1.227 × VO_2_ at the VAT [mL/kg/min]) | 0.907 | 0.895 |
| (5) VO_2peak_ [mL/kg/min] = 20.931 + (-0.098 × age [years]) + (-0.281 × body fat [%]) + (0.761 × OUES/kg) | 0.944 | 0.938 |
| (6) VO_2peak_ [mL/kg/min] = 18.967 + (-0.098 × age [years]) + (-0.254 × body fat [%]) + (-1.455 × active clinical disease) + (0.509 × VO_2_ at the VAT [mL/kg/min]) + (0.542 × OUES/kg) | 0.959 | 0.951 |
| *Steep ramp test WR_peak_* |  |  |
| (1) VO_2peak_ [mL/kg/min] = -4.421 + (8.718 × SRT WR_peak_ [W/kg]) | 0.892 | 0.892 |

Abbreviations: CPET = cardiopulmonary exercise test, OUES = oxygen uptake efficiency slope, VAT = ventilatory anaerobic threshold, VO_2_ = oxygen uptake, VO_2peak_ = oxygen uptake at peak exercise.
